# The Author

**Published:** 1884-11

**Authors:** 


### THE AUTHOR.

The article in the last number of this Journal, entitled "A Visit to the Dentist," was published anonymously because the name of its author was not appended in the manuscript. It should have been credited to Dr. C. S. Stockton, of Newark, New Jersey.

